# Chemical Modifications of Porous Shape Memory Polymers for Enhanced X-ray and MRI Visibility

**DOI:** 10.3390/molecules25204660

**Published:** 2020-10-13

**Authors:** Grace K. Fletcher, Landon D. Nash, Lance M. Graul, Lindy K. Jang, Scott M. Herting, Matthew D. Wilcox, Tyler J. Touchet, Ana Katarina Sweatt, Mary P. McDougall, Steven M. Wright, Duncan J. Maitland

**Affiliations:** 1 Texas A&M University Biomedical Engineering, Bizzell St, College Station, TX 77843, USA; gracekfletch@tamu.edu (G.K.F.); lancegraul58@tamu.edu (L.M.G.); lindy7@tamu.edu (L.K.J.); smherting@tamu.edu (S.M.H.); mattwilcox@tamu.edu (M.D.W.); tytouchet@tamu.edu (T.J.T.); anasweatt@tamu.edu (A.K.S.); mpmcdougall@tamu.edu (M.P.M.); smwright@tamu.edu (S.M.W.); 2 Shape Memory Medical Inc., Santa Clara, CA 95054, USA; landon@shapemem.com; 3 Texas A&M University Electrical and Computer Engineering, Bizzell St, College Station, TX 77843, USA

**Keywords:** porous polymers, shape memory polymers, polyurethanes, X-ray visibility, MRI visibility, biomaterial

## Abstract

The goal of this work was to develop a shape memory polymer (SMP) foam with visibility under both X-ray and magnetic resonance imaging (MRI) modalities. A porous polymeric material with these properties is desirable in medical device development for applications requiring thermoresponsive tissue scaffolds with clinical imaging capabilities. Dual modality visibility was achieved by chemically incorporating monomers with X-ray visible iodine-motifs and MRI visible monomers with gadolinium content. Physical and thermomechanical characterization showed the effect of increased gadopentetic acid (GPA) on shape memory behavior. Multiple compositions showed brightening effects in pilot, T_1_-weighted MR imaging. There was a correlation between the polymeric density and X-ray visibility on expanded and compressed SMP foams. Additionally, extractions and indirect cytocompatibility studies were performed to address toxicity concerns of gadolinium-based contrast agents (GBCAs). This material platform has the potential to be used in a variety of medical devices.

## 1. Introduction

Shape memory polymers (SMPs) are a class of polymeric materials with an ability to change geometry in response to external stimuli. Thermoresponsive SMP materials actuate across a characteristic transition temperature (T_trans_) that is based on polymeric structure. Temperature elevation above the polymer’s T_trans_ enables deformation into a secondary geometry. Maintaining the deformation while cooling below T_trans_ temporarily programs this secondary shape. The unconstrained material will return to the primary geometry when heated back above the T_trans_. This behavior enables a variety of biomedical applications such as conformal bone defect grafts, self-tightening sutures, and devices for minimally-invasive procedures [1,2,3].

Porous polymeric scaffolds are useful in a variety of applications, particularly those requiring tissue ingrowth, as the porous network and large surface area promote cellular infiltration, attachment, and rapid clot formation [4,5,6]. A class of biocompatible thermoset polyurethane SMPs using aliphatic isocyanates that can be gas-blown into low density, porous morphologies was originally envisioned for use in biomedical applications [7,8]. To utilize these properties, these foams and modifications thereof have been implemented in variety of embolic device designs [5,6]. In this case, the shape memory behavior coupled with the porous foam morphology enables minimally invasive delivery of medical devices as the foams can be compressed to low diameters for catheter-guided delivery [9]. However, a major limitation of the SMP materials is that they are not visible on medical imaging modalities.

Many medical procedures are guided by or monitored using X-ray fluoroscopy. Since polymers lack the high density required for radiopacity, there has been extensive work to achieve X-ray visibility through a variety of chemical and physical additive approaches [10,11,12]. Use of nanoparticle and microparticle fillers to improve opacity in the SMP foams has been explored [13,14,15,16,17], but these composite methods can alter bulk thermal and mechanical properties. Another possible approach is chemically incorporating a radiopaque monomer. Recently, Lex et al. reported on polyesters with enhanced X-ray contrast derived from custom iodinated monomers [18].

This work will build upon previous work by Nash et al. who achieved adequate visualization of thermoset SMP foams by chemically incorporating iodine-containing motifs [19]. The radiopaque monomer used in this work is 5-amino-2,4,6-triiodoisphthalic acid (ATIPA) which contains a triiodobenzene ring with an amine group and two carboxylic acid groups for incorporation into the polyurethane network. Triiodobenzene iodine motifs are commonly used in biomedical contrast agents such as Iohexol and Iopamidol due to their absorption of X-rays. While X-ray is a common medical imaging modality, it does necessitate patient exposure to ionizing radiation which can be a concern for pediatric populations and patients requiring imaging often. Furthermore, it does not provide physicians with the same level of dynamic anatomical information as other imaging modalities.

Magnetic resonance imaging (MRI) is a medical imaging modality that does not expose patients to ionizing radiation and produces images with superior soft tissue contrast. MRI visibility in polymers is a topic being explored in biomaterial research. The best strategy for imparting MRI visibility into materials while avoiding device heating during imaging is to generate positive contrast with a passive technique. The most common manifestation of this approach involves MR contrast agents, most often gadolinium-based contrast agents (GBCAs), incorporated into the device in some manner to utilize the T_1_-shortening effects of these agents [20,21]. Younis et al. grafted a GBCA onto a poly (methyl methacrylate)-based copolymer which was ultimately utilized as a coating for a polypropylene mesh [22]. Other approaches for MR visibility in medical devices include incorporating other paramagnetic components; for example, Brocker et al. investigated the use of iron oxide woven into a polypropylene mesh material to achieve MRI visibility in devices [23].

In general, these MRI contrast agents rely on paramagnetic effects to shorten T_1_ relaxation times, decreasing T_1_ saturation effects and leading to increased signal intensity when imaging using T_1_-weighted sequences [24]. Gadolinium is commonly used in this way to generate MRI contrast because it enhances the proton relaxation of surrounding water and its paramagnetism is preserved when complexed with or conjugated to other molecules [24,25]. Weems et al. previously incorporated the monomer diethylenetriaminepentaacetic (DTPA) acid gadolinium (III) dihydrogen salt hydrate also known as gadopentetic acid (GPA) into thermoset SMP foams based on trimethyl hexamethylene diisocyanate (TMHDI) [26]. The carboxylic acid groups on this monomer allow for incorporation into the polymer backbone. This approach also utilized the same structure of gadolinium chelate in the commercially available GBCA Magnevist, which provides a reference for acceptable, nontoxic levels of gadolinium.

This paper will explore a SMP material with both X-ray and MR visibility that could be modified for multiple applications. A few groups have previously successfully modified polymers containing dual-modality contrast, including Goodfriend et al. who synthesized a bioresorbable polyester named poly(gadodiamide fumaric acid) that is X-ray-visible in its liquid coating form and MRI-visible in nanoparticle form [27]. Weems et al. also physically incorporated iron oxide nanoparticles for enhanced SMP visibility on both X-ray and MR imaging modalities [26].

The proposed material platform could be used in many applications requiring guided delivery and follow-up monitoring. For example, X-ray fluoroscopy could be used in device delivery, but MRI could be used for post-procedural monitoring which would reduce the lifetime exposure to ionizing radiation and allow physicians to see soft tissue features. Combining the approaches established by Nash (ATIPA) and Weems (GPA) will create a new porous SMP with X-ray and MR visibility imparted by chemical modifications.

**Figure 1:**
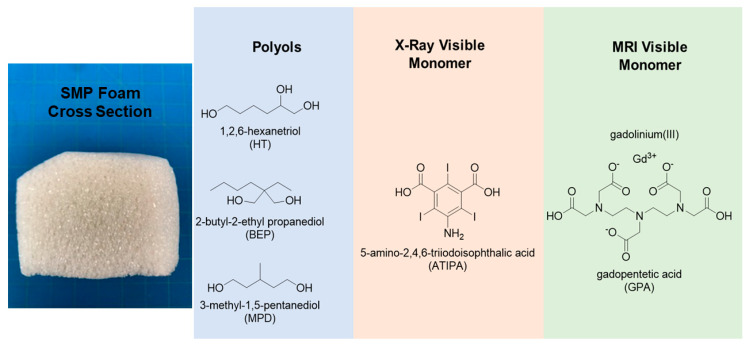
Left—Photograph of the cross section of a synthesized foam at current reaction scale. Right—Hydroxyl or carboxylic acid containing monomers and their role in synthesis of a porous shape memory polymer (SMP) foam with dual contrast on X-ray and MRI modalities.

The material used in this work is based on Nash et al., and is an amorphous thermoset SMP where the glass transition temperature (T_g_) is controlled by tuning the material crosslink density [19]. The addition of GPA to this material system will increase T_g_ due to increased polymer network rigidity and crosslinking. Porosity is an important feature of these shape memory polymers and must be balanced with the correct amount of contrast monomers to ensure visibility in both compressed and expanded forms of the foam. Figure 1 shows the role of each monomer in the material system. Morphological, chemical, and thermomechanical characterizations were performed on multiple compositions. X-ray and MR imaging pilot studies were performed to verify visibility. Furthermore, extractions under simulated use conditions and indirect cytocompatibility studies were conducted to assess toxicity.

## 2. Results

The SMP foam compositions synthesized for the studies were named according to the convention delineated in Table 1. The compositional names arise from the amounts of ATIPA (X-ray-visible monomer) and GPA (MR-visible monomer) in each composition. These are reported as mol% of functionalities reactive with isocyanates (OH, COOH, NH_2_). The isocyanate used in this work is hexamethylene diisocyanate (HDI).

### 2.1. Physical Characterization

Physical characterization included density and pore size measurements. Table 2 contains the SMP foam densities and pore sizes for all compositions made. Pores were measured in the axial and transverse directions.

**Figure 2:**
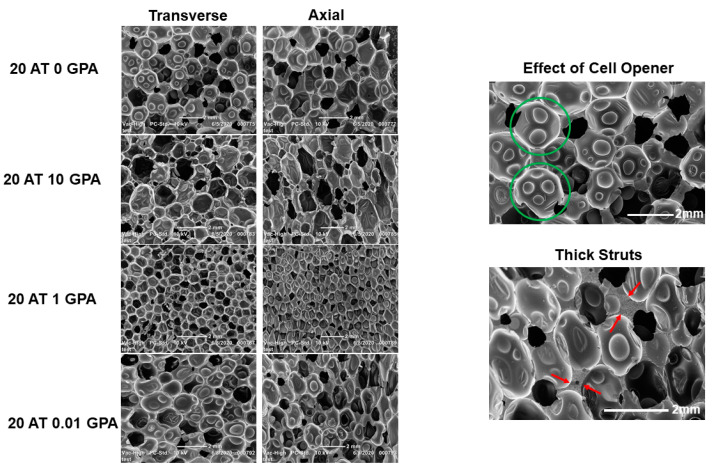
Left—SEM Images of selected foam compositions highlighting pore size and morphology (scale bar 2 mm for all images). Right—zoomed in SEM images highlighting unique morphological features. The green circle denotes the thinning of pore membrane due to the addition of cell opener in the 20 AT 0 GPA composition. The red arrows denote thick struts in composition 20 AT 0.01 GPA.

The SEM images of the foam (Figure 2) show the effect of the cell opener as well as the thick struts in the ATIPA GPA foams. Pore size and morphology varied in compositions due to pre-polymer viscosity and amount of GPA. Foams with larger pores have elongated pores in the axial (foaming) direction. Thick struts were visible in both the 20 AT 0.01 GPA and 20 AT 0.001 GPA compositions.

**Figure 3:**
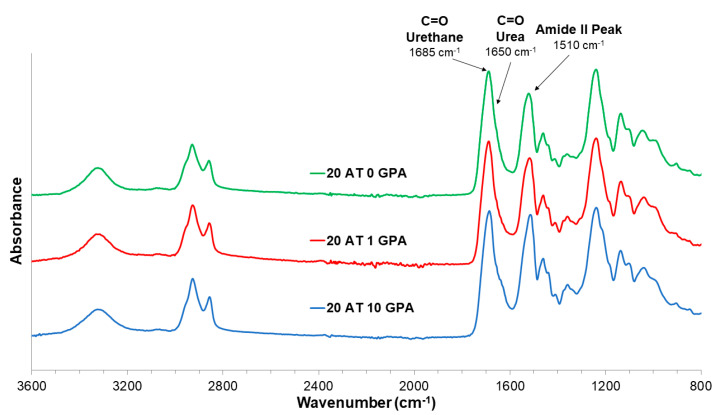
ATR-FTIR spectroscopy results for a selection of compositions with peaks of interest identified (C=O Urethane 1685 cm^−1^, C=O Urea 1650 cm^−1^, Amide II 1510 cm^−1^).

Figure 3 shows the ATR-FTIR spectroscopy for selected compositions. A strong urethane C=O peak is present at 1685 cm^−1^ for all compositions, so spectra were normalized to this peak. Compositions exhibit a urea C=O shoulder at 1650 cm^−1^ from the primary amine on ATIPA’s reaction with isocyanate. The 20 AT 10 GPA composition has the strongest amide II peak at 1510 cm^−1^ due to higher carboxylic acid content in the synthesis from the GPA monomer.

### 2.2. Thermomechanical Characterization

**Figure 4:**
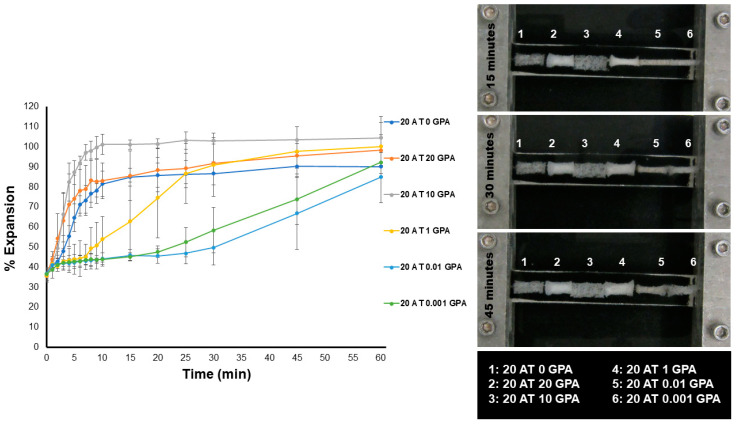
Left—unconstrained expansions of 6 mm diameter foam punches upon exposure to a 37 °C water bath. Images were analyzed every minute for the first 10 min and at 5 min intervals until 30 min, then at 15 min intervals for the remaining time points. Right—images from the water bath at the 15, 30 and 45 min time points to show expansion of foams over time.

Glass transition temperatures (Table 2) increased with increasing GPA content. However, all compositions also demonstrated water plasticized transition temperatures (wet T_g_’s) close to body temperature. Figure 4 summarizes the unconstrained expansion behavior of the foams in a 37 °C water bath. The 20 AT 10 GPA foam had the most rapid expansion, reaching 100% expansion at ~10 min. The 20 AT 0 GPA foam did not reach 100% expansion but also reached its terminal diameter quickly (~15 min). The 20 AT 0.01 GPA and 20 AT 0.001 GPA foams expanded the slowest and did not reach 100% expansion at 60 min. The expansion behavior of the foams seems to be related to both foam density and GPA content increasing hydrophilicity of the foam.

Tensile tests were performed on rectangular foam samples (*n* = 3) affixed to wooden blocks. The ultimate tensile strength (UTS) was calculated from the peak stress on a stress–strain curve (Table 3). UTS and stiffness increases correlate to increasing SMP foam density, except in the case of the 20 AT 20 GPA formulation. This is likely due to increased loading of GPA.

### 2.3. Magnetic Resonance Imaging

**Figure 5:**
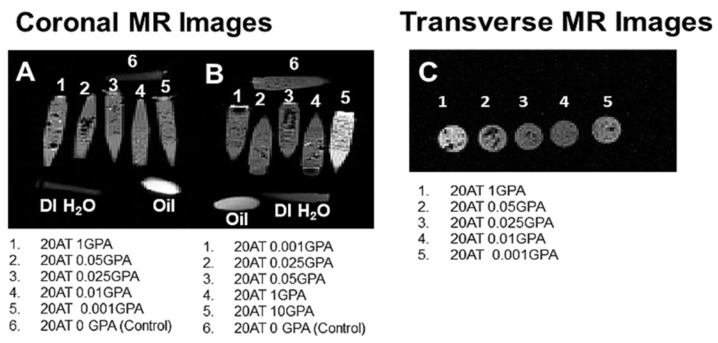
MR images using T_1_-weighted parameters (TE = 30 msec, TR = 500 msec) are shown: (**A**,**B**) Coronal MR images of compositions as labelled with oil and water controls; (**C**) Transverse MR images of compositions as labelled.

The T_1_-weighted MR images were taken in both coronal and transverse planes. Low GPA concentration foams (Figure 5A) did not enhance foam visibility in the coronal view relative to the positive oil control and negative DI water control. However, the 20 AT 10 GPA foam (#5 on Figure 5B) showed a marked brightening effect around the foam and the vial in the coronal view. This intensity exceeds that of the fiducial control (oil capsule) and the other foams in the same image. There is a slight brightening effect of the 20 AT 1 GPA (#1 on Figure 5C) foam in the transverse view. The 20 AT 20 GPA composition is excluded from MR images because we observed darkening due to high concentrations of gadolinium.

### 2.4. X-ray Imaging

**Figure 6:**
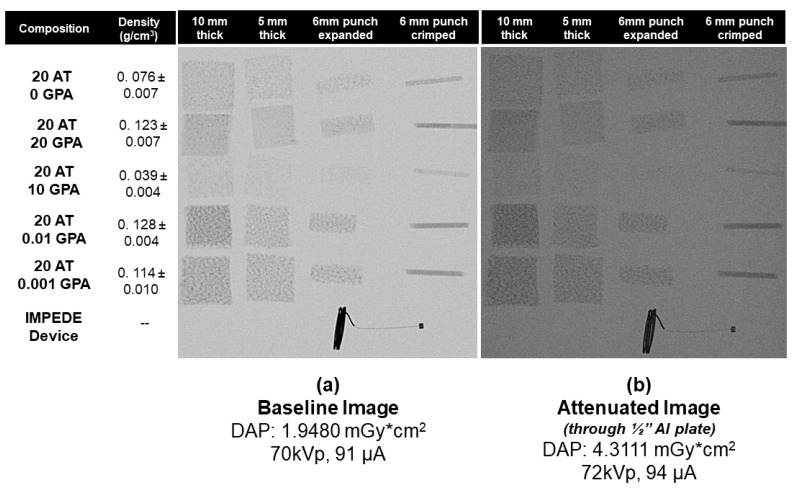
Selected SMP foams with 20 mol% ATIPA and varying amounts of GPA imaged on OrthoScan C-arm. The left image (**a**) was imaged directly to serve as a baseline image. The right image (**b**) was performed through a ½” Al plate to simulate the attenuated image when imaging through bone. Foam samples in columns are labeled with thickness for cubes and crimp state of 6 mm diameter cylindrical biopsy punch samples.

**Figure 7:**
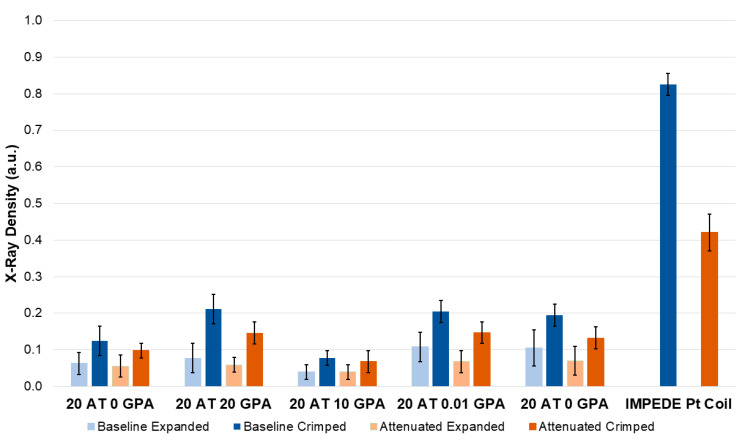
The 6 mm cylindrical foam punches of multiple compositions in expanded and crimped form for each of the X-ray images were analyzed to determine their relative opacity: (Blue) Baseline X-ray image; (Orange) Attenuated X-ray image taken through ½” aluminum plate.

The X-ray images were performed on multiple foams with varying densities. The images were taken both directly and through a ½” aluminum plate to mimic attenuation from bone (e.g., skull) [28]. The X-ray images obtained (Figure 6) were analyzed by measuring the pixel intensity of 60 points on the sample of interest (Figure 7). This was performed after a background removal processing step in ImageJ. The ATIPA-GPA foam X-ray pixel intensity values were compared to those for the platinum coil (Pt Coil) of the IMPEDE device (Shape Memory Medical Inc, Santa Clara, CA, USA), which was analyzed as a control for the opacity of metallic device components.

Figure 7 shows the relative X-ray densities of the 6 mm foam samples in crimped and expanded forms. The images taken with a ½” aluminum plate on top all have lower X-ray densities than their raw image counterparts, which is expected due to X-ray attenuation. Foam density was the largest factor in X-ray opacity since all compositions contained the same amount of ATIPA monomer (20 mol%). The 20 AT 20 GPA, 20 AT 0.01 GPA, and 20 AT 0.001 GPA compositions are the highest density materials and show the greatest X-ray density in both the baseline and attenuated images. The porous morphology of the 20 AT 0.01 GPA and 20 AT 0.001 GPA foams is visible in the X-ray images due to thicker struts.

### 2.5. Mass Spectroscopy of Extractables

Extractions were performed in DI water and 50% ethanol extraction vehicles to identify the amount of extractable and leachable gadolinium under simulated use conditions. The gadolinium concentrations for each composition and extraction condition are reported in Table 4. The highest reported extraction concentration of 7510 ng/mL was reported for the 20AT 20 GPA composition extracted in 50% ethanol, equivalent to a total extracted weight of 75.1 mg for the sample. This is approximately 8 times more gadolinium than the equivalent extraction in DI water.

### 2.6. Indirect Cytocompatibility

**Figure 8:**
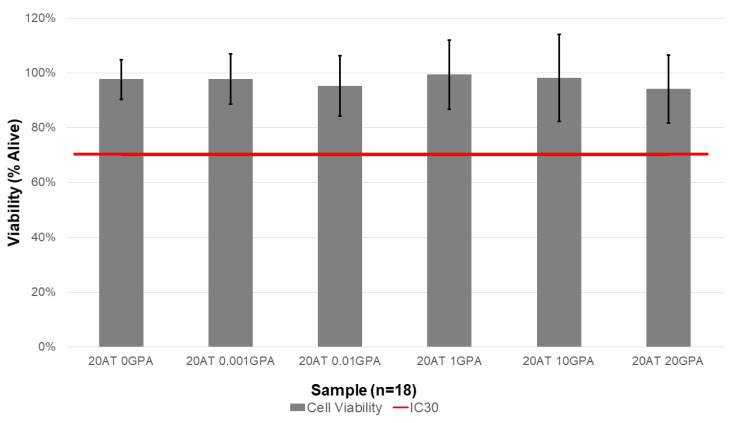
Cell viability was calculated from fluorescence in resazurin assay performed upon 3T3 cell exposure to media extracts with compositions of SMP foams. Extractions and cytocompatibility assays (*n* = 6 wells for each assay) were repeated in triplicate for a total sample size of 18. The red line indicates IC30 or threshold where 70% of cells are alive. All compositions were above the IC30 threshold.

Media extracts were exposed to 3T3 cells in order to determine the compositions’ cytocompatibility and results are displayed in Figure 8. Cell viability was determined using a resazurin assay and calculated from Equation (3). While the composition with the highest GPA content (20 AT 20 GPA) did have the lowest cell viability, all compositions were above the IC30 threshold indicated by the red line in Figure 8.

## 3. Discussion

In general, as GPA content decreased, the density of the polymers increased. This is due to the GPA monomer’s carboxylic acid groups, which acted as an additional blowing agent due to the generation of carbon dioxide upon reacting with isocyanates. The exception to this trend was the 20 AT 20 GPA composition with a higher density. This level of GPA in the 20 AT 20 GPA composition increased the reactive mixture’s viscosity, which decreased the average pore size and increased ultimate material density, despite increased carboxylic acid chemical blowing.

Glass transition temperatures (Table 2) increased with increasing GPA content. This trend was also observed in Weems et al. [26] and is due to restricted chain mobility in the polymeric structure with increasing rigid GPA monomer incorporation. However, all compositions demonstrated water plasticized transition temperatures close to body temperature. The materials’ high dry T_g_’s are beneficial in terms of medical device shipping and storage conditions to maintain secondary geometries without premature actuation.

The unconstrained expansion behavior of the foams in a 37 °C water bath (Figure 4) was more affected by material density and pore size than composition T_g_. The lowest density 20 AT 10 GPA foam was the quickest to expand. This is due to higher density materials contributing a more significant physical diffusion barrier to moisture plasticization. Moisture plasticization depresses the transition temperatures due to disruption of hydrogen bonding between adjacent urethane and urea linkages, resulting in increased segmental mobility within the polymer network. All compositions had moisture plasticized transitions close to body temperature, therefore linking expansion rate to plasticization rate.

The pilot MR imaging studies provided a range of acceptable concentrations of gadolinium. Gadolinium causes shortening of both T_1_ and T_2_. When using T_1_-weighted imaging sequences, the T_1_ shortening manifests as the clinically desired image brightening, while T_2_ shortening will result in image darkening. Depending on the concentration of gadolinium used, one of these effects will be dominant. The 20 AT 20 GPA composition had too high of a concentration of gadolinium and T_2_ effects dominated, so it was excluded from further study. While brightening was seen for the 20 AT 10 GPA and 20 AT 1 GPA compositions, the pilot MR images are somewhat limited since all materials were imaged in vials containing DI water. Although DI water is not entirely representative of T_1_ and T_2_ tissue MR properties, general trends of increased brightening for GBCA-incorporated compositions should still be clinically relevant for T_1_-weighted imaging sequences. In general, visibility of any foam is expected to be dependent on the surrounding tissue properties as well as the exact imaging parameters used. Future imaging studies should be conducted in animal tissues using a variety of imaging parameters to account for tissue variations in MR and X-ray imaging properties.

While reaction conditions were kept constant between compositions, the differences in viscosity of monomer solution and amount of the GPA monomer resulted in different material density and pore sizes. Since material density played a large role in material performance (expansion, tensile strength, and X-ray visibility), it will likely be tuned for specific performance outcomes depending on the chosen application. For example, larger pore and lower density foams will expand faster, but have lower mechanical strength and require a higher loading of contrast agents for visibility, whereas higher density foams will expand slower, but have superior mechanical strength and visibility. Ideal material density can be tailored in each composition by changing the amount of physical blowing agent and reactive mixture viscosity.

The extractions of the 20 AT 20 GPA composition using a 50% ethanol extraction vehicle in simulated use conditions had the highest amount of extracted gadolinium, however, it was well below the maximum doses administered for adult and pediatric populations. Magnevist is prepared at a concentration of 469 mg/mL and administered at a maximum dose of 0.6 mL/kg for adult patients and 0.4 mL/kg for pediatric patients. Using these dosing guidelines, almost 19 devices with a similar footprint (6 mm diameter, 25 mm length cylinder) as the extracted foam sample could be delivered to a 10 kg pediatric patient. In adult patients of 50 kg, approximately 187 devices could be delivered while being in the dosing safety threshold (CDC currently reports an average weight of women and men in the US of ~77 kg and 89 kg respectively).

Indirect cytocompatibility study results complement the extraction ICP-MS data. The 20 AT 20 GPA composition had the lowest average cell viability (94.1 ± 12.3%). However, all compositions were above the IC30 threshold. Gadolinium is toxic because a free Gd^3+^ ion can interfere with normal biological processes due to its similarity to the Ca^2+^ ion [29]. Chelated versions of gadolinium are used to prevent this, but future in vivo studies should carefully monitor accumulation of Gd^3+^ in tissue as well as renal function.

The approaches for imparting X-ray and MRI visibility to SMP foams through chemical modification described herein could be translated to many material systems. Additionally, SMP foam properties could be tuned for specific device requirements. These foams could be incorporated into current embolic device designs to reduce or eliminate metallic components used for visualization. The foam itself being visible provides more information about the expansion and volume-filling properties of the devices. An example of a new application amenable to the developed material system is a biopsy sealing device. The benefit of the X-ray and MRI visibility for this application would be that the device could be imaged during delivery and the polymer plug could act as a fiducial to mark the tract where the biopsy was taken from.

## 4. Materials and Methods

### 4.1. Materials

All chemicals were used as purchased unless otherwise indicated. 3-methyl-1,5-pentanediol, 2-butyl-2-ethyl propanediol, 1,2,6-hexanetriol, 5-amino-2,4,6-triidoisosphthalic acid (95%) and gadopentetic acid (97%) were all purchased from Sigma Aldrich. Hexamethylene diisocyanate was purchased from TCI. The cell opener Tegostab B8523 and surfactant DC1990 were provided by Evonik. Tetrahydrofuran was from EMD Millipore and was stored over molecular sieves. Isopropanol was purchased from VWR. Enovate was purchased from Honeywell.

### 4.2. SMP Foam Synthesis

All foam compositions used in the study are listed in Table 1. Foams were synthesized using a classic hydroxyl (OH) and isocyanate (NCO) polyurethane reaction scheme. The hydroxyl components used were 3-methyl-1,5-pentanediol (MPD), 2-butyl-2-ethyl propanediol (BEP), and 1,2,6-hexanetriol (HT). The isocyanate component was hexamethylene diisocyanate (HDI). The X-ray-visible monomer used was 5-amino-2,4,6-triiodosphthalic acid (ATIPA). The MRI-visible monomer was diethylenetriaminepentaacetic acid gadolinium (III) dihydrogen salt hydrate also known as gadopentetic acid (GPA). All monomers were used as received and premixes were prepared in a glovebox.

The foaming procedure was adapted from Nash et al. to accommodate addition of gadolinium [19]. The hydroxyl components (0.6 molar eq.) of the OH premixes (MPD, BEP, HT, ATIPA) were measured out into a 150 mL Flacktek mixing cup two days before foaming. They were mixed in a Flacktek high speed mixer (Model DAC 150 FVZ-K, Flacktek Inc, Landrum, SC, USA) for 30 s at 3540 rpm and placed in an oven at 50 °C. After 1 h, the cup was mixed again for 30 s and remained in the 50 °C oven overnight.

Similarly, the hydroxyl components (0.4 molar eq.) of the NCO premixes were added to a separate Flacktek mixing cup two days prior to foaming. They were mixed in a Flacktek high speed mixer for 30 s at 3540 rpm and placed in an oven at 50 °C. After 1 h, the cup was mixed again for 30 s and remained in the 50 °C oven overnight. The following day, the contents were mixed another 30 s before adding the diisocyanate equivalents (1.02 molar eq.). The mixture was mixed in a Flacktek high speed mixer for 5 min at 3540 rpm and placed on a shaker plate at room temperature overnight or until the mixture’s viscosity increased to a honey-like consistency.

The surfactant DC1990 (4 wt%) and the cell opener Tegostab B 8523 (0.025 wt%) were added to the NCO premix prior to foaming and mixed for 30 s. GPA was added to the OH premix and mixed for 1 min immediately before foaming. The OH premix was added to the NCO premix and mixed for 30 s. A physical blowing agent, Enovate, was added to that mixture and mixed for 15 s. The cup was moved to an oven at 90 °C and allowed to cure for 20 min. The foam was allowed to cool in a fume hood and the skin was removed with a razor blade before an overnight post-cure in a 50 °C oven.

Foam samples were placed in a jar and submerged in DI water and sonicated for three 30 min cycles to rinse before adding isopropyl alcohol (IPA) in a 20:1 IPA:foam volume ratio. The jars were subjected to three 30 min intervals in a sonication bath, switching out IPA between intervals. They were dried overnight at 100 °C in a vacuum oven and stored with desiccant before characterization took place unless otherwise noted.

### 4.3. Physical Characterization

#### 4.3.1. Density

Cubes from each foam block were used for density measurements. The dimensions of the block were measured (*n* = 3) with calipers and volume was calculated. Mass of the blocks was measured on a balance (*n* = 3). Density was calculated using averaged mass and volumes for the samples.

#### 4.3.2. Pore Morphology

Foam slices were cut in the axial and transverse directions in the middle of the foam, mounted on carbon tape affixed to an imaging stub, dried, and sputter coated. Slices were imaged using a JEOL JCM-5000 Neoscope scanning electron microscope (JEOL USA Inc., Peabody, MA, USA). 30 pores were measured per slice direction using ImageJ software.

#### 4.3.3. Fourier Transform Infrared Spectroscopy (FTIR)

Foam samples were cut and compressed to be measured using a Bruker Spectrometer (Bruker, Billerica, WA, USA). Spectra were obtained via a germanium attenuated total reflectance (ATR) probe. Thirty-two background scans were performed prior to each sample measurement. Samples were measured using 64 scans and the resulting spectra were corrected for atmospheric compensation using Bruker OPUS software and exported to Excel where they were normalized to the urethane peak.

### 4.4. Gel Fractions

Gel fraction was performed on cleaned and dried foams to determine the extent of crosslinking. The initial cleaning step removes excess surfactant and catalysts. Original dry weight was measured using a balance (Mettler Toledo, Columbus, OH, USA). Foam blocks were incubated in tetrahydrofuran (THF) for 3 days at 50 °C. Foams were dried at 50 °C under vacuum for 2 days and measured to get the final weight. Gel fraction (G_f_) was calculated using the equation:

(1) $$\frac{m_{1}}{m_{0}}\times \;100\%\;=\;G_{f}$$

where *m*_0_ is original clean foam dry mass and *m*_1_ is dry foam mass after incubation in THF.

### 4.5. Thermomechanical Characterization

#### 4.5.1. Differential Scanning Calorimeter (DSC)

Differential scanning calorimetry measurements were used to determine the glass transition temperature (T_g_) of wet and dry samples. Measurement were obtained using a TA Q200 Differential Scanning Calorimeter (TA Instruments, New Castle, DE, USA). Hermetically sealed aluminum Tzero (Switzerland) pans with a hole poked in the top were used for all samples and goal foam sample weight was 5–10 mg. The cycle used for dry samples (Dry T_g_) was first equilibrated at −40 °C for 5 min, heated to 120 °C (40 °C/min) and cooled to −40 °C. It was then reheated again at 10 °C/min to 120 °C. The second heating curve was analyzed using TA Universal Analysis software to determine dry T_g_. Wet T_g_ foam samples were incubated in a 50 °C water bath for 30 min and pressed between Kimwipes to remove moisture prior to running. The cycle for wet samples was equilibrated at −40 °C for 5 min, with a single heating cycle that ramps to 100 °C at 10 °C/min. The wet T_g_ was determined from the inflection point on the heating curve using TA Universal Analysis software.

#### 4.5.2. Unconstrained Expansions

After cleaning and drying, 6 mm cylindrical biopsy punches of foams (*n* = 3) were threaded over a wire and initial images were taken. The punches were radially crimped using Machine Solutions SC250 radial crimper heated to 100 °C. The crimped foams were allowed to relax at room temperature in a desiccated container for 24 h before studying their expansion behavior. Images of crimped foams were taken to determine initial crimped diameter. A water bath was heated to 37 °C and pictures were taken every minute until the 10 min time point, then images were taken every five minutes until 30 min. All images were analyzed by measuring five points on each foam punch at every time point using ImageJ. Results were reported as percent expansion with standard deviation.

#### 4.5.3. Tensile Testing

Rectangular foam samples were cut to approximately 25 mm × 15 mm × 3 mm and epoxied to wooden blocks at the short end. Epoxy was allowed to cure overnight and samples were stored under vacuum in a bell jar for at least 24 h prior to tensile testing to ensure ambient moisture did not affect mechanical properties. An MTS Insight 30 Universal Tensile Tester (MTS Systems Corp, Eden Prairie, MN, USA) with a 50 N load cell was used in tensile testing. Clamps were tightened on the wooden blocks at either end of the sample and machine was zeroed. The protocol used was a strain to failure at 5 mm/min constant strain rate.

### 4.6. Magnetic Resonance Imaging (MRI)

Pilot magnetic resonance imaging (MRI) validation studies of the SMP foams were performed at the Magnetic Resonance Systems Lab at Texas A&M University. Cylindrical foam samples (6 mm diameter) were placed in DI water in microcentrifuge tubes and a fish oil tablet was used as a positive control for images. High resolution multi-slice T_1_-weighted images (TR 500msec, TE 30 msec) were acquired on a 4.7T Varian Inova scanner using a standard spin-echo pulse sequence. Transverse images were taken with a 30 mm × 60 mm field-of-view and 64 × 128 matrix size for 469 µm resolution in both x (left-right) and y (anterior-posterior) dimensions with a slice thickness of 1 mm. For coronal images, a 120 mm × 80 mm field-of-view and 256 × 128 matrix size were used for 469 µm resolution in the z (head-foot) dimension and 625 µm resolution in the x dimension with a slice thickness of 1 mm. All other parameters were kept constant throughout all acquisitions. The raw data acquired from the scanner were imported to Matlab^®^ for reconstruction and display of all images.

### 4.7. X-ray Imaging

Foam samples were cut into blocks (~10 mm × 10 mm) of 10 mm and 5 mm thicknesses and cylinders of 6 mm diameter. All samples were arranged according to composition and imaged using OrthoScan C-arm system (Mobile DI Model 1000-0005, Orthoscan, Scottsdale, AZ, USA). There were 6 mm diameter cylindrical prototype devices made as well in crimped and expanded forms. IMPEDE device (Shape Memory Medical, Santa Clara, CA, USA) containing a Pt coil and marker band was used as a positive control. Samples were also imaged through a ½” aluminum plate which served as a bone analog with respect to X-ray attenuation [19]. Images were converted to 8-bit grayscale, processed to remove the background, and analyzed using ImageJ to determine X-ray density. Sixty measurements were taken using the multi-point selector yielded values between 0 (black) and 255 (white). X-ray density was calculated from using the equation

(2) $$X-Ray\;Density=\frac{255-\left(Image\;J\;Pixel\;Intensity\;Value\right)}{255}$$

such that a density of 1 would correlate to an entirely black image.

### 4.8. Mass Spectroscopy of Extractables

The extraction conditions chosen were simulated use, batch extraction with agitation. Cylindrical (6 mm diameter × 25 mm length) SMP foam samples were incubated in 10 mL of extraction solution at 37 °C in an incubation shaker at 90 rpm. DI water was used as the polar extraction solution and a 50/50 water/ethanol solvent mixture was used as the non-polar extraction solution. After 72 h, samples were removed and 1% nitric acid was added to solutions to ensure metallic particle stability before samples were analyzed using a Perkin Elmer NexION 300D inductively coupled mass spectroscopy (ICP-MS) instrument (Perkin Elmer Inc., Shelton, CT, USA) in the Elemental Analysis Laboratory at Texas A&M University.

### 4.9. Indirect Cytocompatibility

Using sterile biopsy punches in an aseptic environment, foams were cut into 8 cm diameter discs with 2 mm thickness. The surface area of the discs was calculated based on the pore size of each foam composition as previously performed by Weems et al. [30]. Foams were submerged in complete cell culture media for extraction at a ratio of 3 cm^2^ foam per 1 mL of media for 72 h at 37 °C in a rotating incubator. An additional tube with media only was included as a cytocompatible control. Complete cell culture media was made from Dulbecco’s Modified Eagle Medium (DMEM, MilliporeSigma, St. Louis, MO, USA) and supplemented with 10% newborn calf serum (NCS, MilliporeSigma, St. Louis, MO, USA), 1% penicillin-streptomycin (P/S, VWR, Radnor, PA, USA) solution, and 0.1% fungizone (VWR, Radnor, PA, USA).

Balb/3T3 cells, clone A31 (3T3s, ATCC, Manassas, VA, USA) were thawed and passed at least once prior to plating for the cytocompatibility assay. All incubation periods were done in a humidified incubator at 37 °C with 5% CO_2_. Complete cell culture medium used for 3T3 culture and the assays was the same as described above for the extraction. 3T3s were harvested with trypsin and seeded in 96 well plates at 7500 cells per well. After a 24 h incubation, cell morphology and distribution were assessed, and images were acquired of each well using a Biotek Cytation5 Imaging Reader (Biotek, Winooski, VT, USA). The media was then removed and replaced with media from the foam extractions or control. The cells were incubated with these treatments for 48 h, then cell morphology and distribution were assessed, and additional images were acquired of each well using the Cytation5. Finally, cell viability was measured indirectly using a resazurin assay to assess relative metabolic activity. Cells were incubated with 5% resazurin in complete media for 3 h, then the fluorescence was measured using an excitation wavelength of 560 nm and an emission wavelength of 590 nm on a Tecan Infinite M200 Pro Plate Reader (Tecan, Morrisville, NC, USA). Cell viability was calculated using the following equation:

(3) $$\mathrm{Cell}\;\mathrm{Viability}\;\left(X\right)=\frac{{\mathrm{RFU}}_{\frac{560}{590}}\left(X\right)-{\mathrm{RFU}}_{\frac{560}{590}}\left(\mathrm{blanks}\right)}{{\mathrm{RFU}}_{\frac{560}{590}}\left(\mathrm{Untreated}\;\mathrm{Control}\right)-{\mathrm{RFU}}_{\frac{560}{590}}\left(\mathrm{blanks}\right)}$$

where X is any treatment group and RFU is relative fluorescent units (i.e., fluorescence intensity). Extractions and cytocompatibility assays were repeated in triplicate.

## Figures and Tables

**Table 1:** Hydroxyl components of shape memory polymer compositions synthesized for use in all studies.

Composition	ATIPA (eq%)	GPA (eq%)	MPD (eq%)	BEP (eq%)	HT (eq%)
20 AT 0 GPA	20	0	40	20	20
20 AT 20 GPA	20	20	20	20	20
20 AT 10 GPA	20	10	23.3	23.3	23.3
20 AT 5 GPA	20	5	25	25	25
20 AT 2.5 GPA	20	2.5	25.8	25.8	25.8
20 AT 1 GPA	20	1	26.3	26.3	26.3
20 AT 0.05 GPA	20	0.05	26.7	26.7	26.7
20 AT 0.025 GPA	20	0.025	26.7	26.7	26.7
20 AT 0.01 GPA	20	0.01	26.7	26.7	26.7
20 AT 0.001 GPA	20	0.001	26.7	26.7	26.7

**Table 2:** Physical and thermomechanical properties of SMP foams. Measurements are reported as mean ± standard deviation for the indicated sample size in top row.

Composition	Density (g/cm^3^)	Pore Sizes (µm)	Dry T_g_ (°C)	Wet T_g_ (°C)	Gel Fraction (%)
	*n* = 3	*n* = 30	*n* = 3	*n* = 3	*n* = 3
20 AT 0 GPA	0.076 ± 0.007	Axial 1447 ± 344 Trans 1523 ± 329	47.5 ± 0.9	32.1 ± 1.6	97.2 ± 0.3
20 AT 20 GPA	0.123 ± 0.007	Axial 702 ± 108 Trans 711 ± 118	66.8 ± 1.0	39.2 ± 1.1	98.8 ± 0.3
20 AT 10 GPA	0.039 ± 0.004	Axial 1853 ± 505 Trans 1493 ± 311	66.9 ± 0.3	37.1 ± 0.9	97.5 ± 0.1
20 AT 1 GPA	0.093 ± 0.003	Axial 920 ± 199 Trans 744 ± 122	64.0 ± 0.9	36.0 ± 1.0	98.0 ± 0.1
20 AT 0.01 GPA	0.128 ± 0.004	Axial 1408 ± 93 Trans 798 ± 69	59.8 ± 0.5	36.4 ± 0.7	98.6 ± 0.3
20 AT 0.001 GPA	0.114 ± 0.010	Axial 1211 ± 364 Trans 1051 ± 229	54.9 ± 1.1	37.8 ± 1.8	98.3 ± 0.4

**Table 3:** Ultimate tensile strength (UTS) for compositions with varied GPA content.

Composition	Ultimate Tensile Strength (kPa)
20 AT 0 GPA	356.5 ± 36.3
20 AT 20 GPA	455.2 ± 92.2
20 AT 10 GPA	203.7 ± 41.1
20 AT 1 GPA	751.8 ± 126.8
20 AT 0.01 GPA	927.4 ± 101.0
20 AT 0.001 GPA	1061.1 ± 71.8

**Table 4:** ICP-MS results for the ATIPA-GPA foam samples in different extraction vehicles. Results are displayed as Gd concentration ± uncertainty.^1.^

Composition	DI Water Extract Gd Concentration (ng/mL)	50% Ethanol Extract Gd Concentration (ng/mL)
20 AT 20 GPA	938 ± 47	7510 ± 380
20 AT 10 GPA	259 ± 13	2160 ± 110
20 AT 1 GPA	116 ± 6	419 ± 21
20 AT 0.01 GPA	2.4 ± 0.1	38 ± 2
20 AT 0.001 GPA	<0.1 *	0.30 ± 0.02

^1^ Uncertainties provided at 1 s (67% confidence level); * Quantitation limit.
